# Coactivation sign in fixed dystonia

**DOI:** 10.1016/j.parkreldis.2012.10.014

**Published:** 2013-04

**Authors:** Arpan R. Mehta, James B. Rowe, Michael R. Trimble, Mark J. Edwards, Kailash P. Bhatia, Anette E. Schrag

**Affiliations:** Department of Clinical Neurosciences (Neurology Unit), University of Cambridge, Cambridge, UK; Department of Clinical Neurosciences (Neurology Unit), University of Cambridge, Cambridge, UK; Medical Research Council Cognition and Brain Sciences Unit, Cambridge, UK; Behavioural and Clinical Neuroscience Institute, Cambridge, UK; Department of Neuropsychiatry, Institute of Neurology, London, UK; Institute of Neurology, University College London, London, UK

**Keywords:** Dystonia, Psychogenic movement disorders, Fixed dystonia, Electrophysiology

Laboratory-defined biomarkers can greatly assist the diagnosis and management of psychogenic movement disorders [1]. For example, there are some neurophysiological findings that may support the diagnosis of psychogenic tremor, including coactivation of finger flexors and extensors about 300 ms prior to tremor onset [2]. In contrast, psychogenic dystonia has proven difficult to distinguish from its organic counterpart, and there are no definitive, simple tests [esupp Refs. 1–3] to support clinical diagnostic criteria ([3], esupp Refs. 4–5). Here, we describe an abnormal neurophysiological pattern in a group of patients with fixed lower limb dystonia [4], a typical presentation of psychogenic dystonia ([5], esupp Ref. 5).

We examined surface electromyogram recordings from the right gastrocnemius, right tibialis anterior and left gastrocnemius muscles from patients with either isolated fixed (*N* = 4; 3 females; mean age 38 years; all negative for the *DYT1* gene mutation) or genetically determined and predominantly mobile (*N* = 5; 3 females; mean age 35 years; all positive for the *DYT1* gene mutation) dystonia affecting the right lower limb, and from healthy controls (*N* = 6; 4 females; mean age 30 years). None of the patients with fixed dystonia had complex regional pain syndrome, and none of the diagnoses have been changed over a period of ten years after the recordings were obtained.

Subjects placed their right foot in a customised open plaster-cast that held it in a plantar-flexed, inverted position, with a footplate dynamometer placed underneath their foot. There were three study conditions (“rest”, “posture” and “move”). Each condition included auditory pacing cues (0.125 Hz) from a metronome for a duration of 125 s. In the “move” condition, subjects were asked to make an ipsilateral flexion/extension movement of the ankle every 8 s, in time with the metronome sound. For the “posture” condition, subjects were asked to maintain their right foot in an inverted plantar-flexed posture. In the “rest” condition, subjects were required to rest their feet. A pre-trial period of at least 30 s occurred between the verbal instruction of the forthcoming condition and the onset of the metronome. Each condition was repeated four times per subject, in a counterbalanced order. Subjects gave written informed consent, in accordance with the *Declaration of Helsinki*.

In two patients with fixed dystonia, we found sustained muscle coactivation of ipsilateral gastrocnemius, tibialis anterior, and contralateral gastrocnemius muscles beginning towards the end of the pre-trial period (Fig. 1). It occurred consistently prior to every repetition. Moreover, it occurred before the “rest” condition, as well as before the “posture” and “move” conditions. None of the patients with organic dystonia or controls demonstrated this coactivation pattern. Thus, a coactivation sign, described in patients with psychogenic tremor [2], is also found in a proportion of patients with fixed dystonia.

The underlying pathophysiology of this coactivation phenomenon remains to be established. In intermittent psychogenic tremor it has been postulated to underlie the pathophysiology of the tremor; however, since fixed dystonia is also present between conditions, it is unlikely to be the main mechanism underlying the generation of the abnormal posture. The occurrence of prolonged coactivation even before the “rest” condition suggests that it is distinct from preparation or motor facilitation [esupp Ref. 2]. In fixed dystonia, and possibly also in psychogenic tremor, it may reflect an interaction between attention to the affected limb and the motor system, leading to further muscle contraction. This abnormal electromyographic sign of pre-trial sustained coactivation encourages larger scale investigation of this potential objective biomarker in psychogenic dystonia.

## Financial disclosure for the previous 12 months

A.R.M. was supported by the East of England Multi-Professional Deanery and Foundation School, and received further grant support from the Addenbrooke's Postgraduate Medical Centre of Cambridge University Hospitals NHS Foundation Trust, and Parkinson's UK. J.B.R. is supported by the Wellcome Trust (054016; 088324), Medical Research Council, National Institute for Health Research Cambridge Biomedical Research Centre, James S. McDonnell Foundation, Parkinson's UK and Biotechnology and Biological Sciences Research Council. M.J.E. is supported by an NIHR Clinician Scientist Fellowship. He receives further grant support from Parkinson's UK and the Dystonia Society (UK). He receives royalties from his book: *The Oxford Specialist Handbook of Parkinson's Disease and Other Movement Disorders*, is a member of the editorial board of *Movement Disorders* and has received honoraria for speaking from UCB and the *Movement* Disorders Society. K.P.B. received funding for travel from GlaxoSmithKline, Orion Corporation, Ipsen, and Merz Pharmaceuticals, LLC; serves on the editorial boards of *Movement Disorders* and *Therapeutic Advances in Neurological Disorders*; receives royalties from the publication of *Oxford Specialist Handbook of Parkinson's Disease and Other Movement Disorders* (Oxford University Press, 2008); received speaker honoraria from GlaxoSmithKline, Ipsen, Merz Pharmaceuticals, LLC, and Sun Pharmaceutical Industries Ltd.; personal compensation for scientific advisory board for GSK and Boehringer Ingelheim; received research support from Ipsen and from the Halley Stewart Trust through Dystonia Society (UK), and the Wellcome Trust MRC strategic neurodegenerative disease initiative award (Ref. number WT089698), a grant from the Dystonia Coalition and a grant from Parkinson's UK (Ref. number G-1009). A.E.S. is on the advisory board of Osmotica Pharmaceuticals and has received consultancies from Neurosearch.

## Figures and Tables

**Fig. 1:**
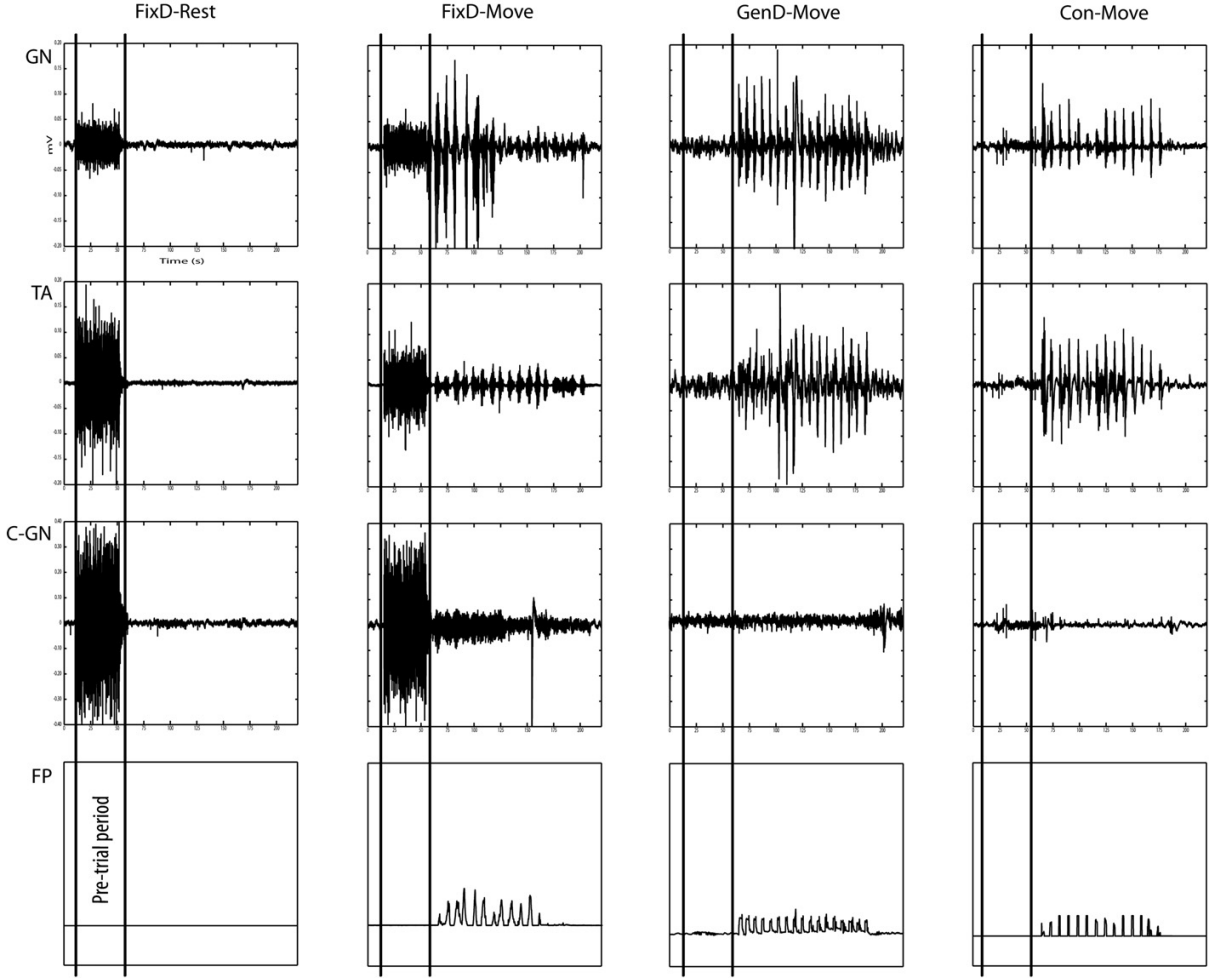
Representative footplate dynamometer (FP) and surface electromyogram recordings from right gastrocnemius (GN), tibialis anterior (TA) and contralateral gastrocnemius (C-GN) muscles from a patient with fixed dystonia of the right lower limb (FixD), demonstrating coactivation of GN, TA and C-GN muscles during the pre-trial period, preceding both “rest” and “move” conditions and not seen between conditions. In contrast, traces depicting “move” trials in a patient with genetically determined dystonia of the right lower limb (GenD) and a control subject (Con) show a lack of coactivation with onset during the pre-trial period. The pre-trial period (marked by vertical lines) spans the time between when the subject was verbally informed of the condition, and the onset of the metronome. Each division on the abscissae denotes 25 s.
