# Supplementary material for: Coactivation sign in fixed dystonia
Source: Parkinsonism Relat Disord. 2013 Apr;19(4):474–6. doi: 10.1016/j.parkreldis.2012.10.014 (PMC3638232; doi:10.1016/j.parkreldis.2012.10.014)
Supplement: Supplementary file 1 [file mmc1.doc]

**[esupp ref 1] Hallett M, Rothwell J. Milestones in clinical neurophysiology. Mov Disord 2011;26:958-67.**

**[esupp ref 2] Hallett M. Physiology of psychogenic movement disorders. J Clin Neurosci 2010;17:959-65.**

**[esupp ref 3] Chen R, Brashear A. A diagnostic test to distinguish psychogenic dystonia from organic dystonia? Neurology 2011;76:590-1.**

**[esupp ref 4] Williams DT, Ford B, Fahn S. Phenomenology and psychopathology related to psychogenic movement disorders. Adv Neurol 1995;65:231-57.**

**[esupp ref 5] Gupta A, Lang AE. Psychogenic movement disorders. Curr Opin Neurol 2009;22:430-6.**
